# Draft Genome Sequences of Lactobacillus helveticus, Lactobacillus fermentum, and Lactobacillus delbrueckii Strains from African Fermented Nono

**DOI:** 10.1128/MRA.01342-19

**Published:** 2020-01-02

**Authors:** Gyu-Sung Cho, Olakunle Fagbemigun, Erik Brinks, Gbenga A. Adewumi, Folarin A. Oguntoyinbo, Charles M. A. P. Franz

**Affiliations:** a Department of Microbiology and Biotechnology, Max Rubner-Institut, Federal Research Institute of Nutrition and Food, Kiel, Germany; b Department of Microbiology, Faculty of Science, University of Lagos, Lagos, Nigeria; c Department of Chemistry and Fermentation Sciences, Appalachian State University, Boone, North Carolina, USA; University of Maryland School of Medicine

## Abstract

The genomes of predominant Lactobacillus helveticus, Lactobacillus fermentum, and Lactobacillus delbrueckii strains from fermented nono were sequenced. The genome sizes were 2.1, 1.9, and 1.7 Mbp, respectively, and the GC contents were 36.5%, 51.5%, and 49.7%, respectively. Annotation revealed some genes for bacteriocin and for the potential production of aroma compounds.

## ANNOUNCEMENT

Nono is a Nigerian fermented milk product that contains essential amino acids, calcium, phosphorus, and vitamins A, C, and E, as well as the vitamin B complex (1). To prepare nono, fresh cow’s milk is left to be fermented by indigenous bacteria in a covered calabash at room temperature (2). A previous study by Obi and Ikenebomeh (3) showed that the pH of nono was below 5.5 after 12 h of fermentation and the number of bacteria had increased to 2.1 × 10^6^ CFU/ml. These bacteria included lactic acid bacteria (LAB) but also could include contaminant pathogens such as Staphylococcus aureus and Escherichia coli strains. In general, for the production of nono products on a more industrial scale, it would be necessary to develop starter cultures. In this study, the genomes of three representative strains of LAB, Lactobacillus helveticus, Lactobacillus fermentum, and Lactobacillus delbrueckii, were isolated from 10-fold serial dilutions of nono samples, of which appropriate aliquots were plated on de Man-Rogosa-Sharpe (MRS) agar plates. Predominant isolates were randomly obtained from plates of the highest dilutions and were identified (results not shown) and sequenced.

For whole-genome sequencing, each strain was cultured separately in MRS broth overnight at 30°C, and the total genomic DNA of each *Lactobacillus* species was extracted using the peqGOLD bacterial DNA kit (Peqlab, Erlangen, Germany), according to the manufacturer’s instructions. The sequencing library was prepared with a Nextera Flex DNA library kit (Illumina, San Diego, CA, USA) and run on the Illumina MiSeq platform (2 × 151 paired ends). A total of 4,043,3471 paired-end and 743 single-end sequence reads were obtained from three samples, with coverages that ranged from 58.8-fold to 67.1-fold. The low-quality reads and adapter sequences were removed using Trimmomatic v0.36 (4). The reads were *de novo* assembled using SPAdes v3.13.1 (5), with a minimum contig length of 500 bp and minimum coverage of 5-fold, and draft genome sequences were annotated using the NCBI Prokaryotic Genome Annotation Pipeline v4.10, with default parameters (6). The genome features and the quality information for *de novo* assembly are presented in Table 1. To identify these strains, the complete 16S rRNA gene sequences were extracted from the PATRIC data set (7) and analyzed in the EzTaxon pipeline, with default parameters (8). The results of the 16S rRNA gene similarity analysis and species identification are shown in Table 1. The whole-genome sequence data showed that all three strains possessed genes that might play a role in yogurt flavor, i.e., genes for an acetate kinase to produce acetate and an acetaldehyde dehydrogenase to produce acetaldehyde (9). Furthermore, the L. helveticus 313 and L. fermentum 317 strains possessed a gene that might play a role in diacetyl production, a citrate lyase gene (9). Lactobacillus helveticus strain 313 possessed the helveticin J gene locus, which may be important in contributing to the safety of the products and to fermentation success. The presence of these genes indicated that these three strains are potentially interesting for use as starter cultures to produce yogurt-like nono with typical aroma compounds; however, this should be investigated further.

**TABLE 1:** *De novo* assembly statistics of three *Lactobacillus* strains from nono

Strain	Total no. of reads	GenBank accession no.	SRA accession no.	Genome coverage (fold)	No. of contigs	No. of coding sequences	Genome size (bp)	*N*_50_ (bp)	GC content (%)	EzTaxon identification (identity [%])
313	1,407,042	WHOE00000000	SRR10332348	60.8	179	2,255	2,103,477	25,324	36.50	*L. helveticus* (99.73)
317	1,332,882	WHOF00000000	SRR10332347	58.8	144	1,950	1,924,745	43,204	51.55	*L. fermentum* (99.86)
328M	1,304,190	WHOG00000000	SRR10332346	67.1	73	1,815	1,785,290	60,623	49.72	*L. delbrueckii* subsp. *bulgaricus* (99.93)

### Data availability.

The whole-genome sequences of *Lactobacillus* sp. strains 313, 317, and 328M have been deposited in DDB/ENA/GenBank under accession numbers WHOE00000000, WHOF00000000, and WHOG00000000, respectively. The raw reads can be found as SRA data with accession numbers SRR10332348 (strain 313), SRR10332347 (strain 317), and SRR10332346 (strain 328M).
